# Macrophage migration inhibitory factor rejuvenates aged human mesenchymal stem cells and improves myocardial repair

**DOI:** 10.18632/aging.102592

**Published:** 2019-12-27

**Authors:** Yuelin Zhang, Wenwu Zhu, Haiwei He, Baohan Fan, Rui Deng, Yimei Hong, Xiaoting Liang, Hongyan Zhao, Xin Li, Fengxiang Zhang

**Affiliations:** 1 Department of Emergency Medicine, Department of Emergency and Critical Care Medicine, Guangdong Provincial People’s Hospital, Guangdong Academy of Medical Sciences, Guangzhou, China; 2 Section of Pacing and Electrophysiology, Division of Cardiology, The First Affiliated Hospital of Nanjing Medical University, Nanjing, China; 3 Department of General Surgery, The Second Affiliated Hospital of Bengbu Medical College, Bengbu, Anhui, China; 4 Clinical Translational Medical Research Center, Shanghai East Hospital, Tongji University School of Medicine, Shanghai, China

**Keywords:** macrophage migration inhibitory factor, mesenchymal stem cells, rejuvenation, senescence, myocardial infarction

## Abstract

The beneficial functions of mesenchymal stem cells (MSCs) decline with age, limiting their therapeutic efficacy for myocardial infarction (MI). Macrophage migration inhibitory factor (MIF) promotes cell proliferation and survival. We investigated whether MIF overexpression could rejuvenate aged MSCs and increase their therapeutic efficacy in MI. Young and aged MSCs were isolated from the bone marrow of young and aged donors. Young MSCs, aged MSCs, and MIF-overexpressing aged MSCs were transplanted into the peri-infarct region in a rat MI model. Aged MSCs exhibited a lower proliferative capacity, lower MIF level, greater cell size, greater senescence-associated-β-galactosidase activity, and weaker paracrine effects than young MSCs. Knocking down MIF in young MSCs induced cellular senescence, whereas overexpressing MIF in aged MSCs reduced cellular senescence. MIF rejuvenated aged MSCs by activating autophagy, an effect largely reversed by the autophagy inhibitor 3-methyladenine. MIF-overexpressing aged MSCs induced angiogenesis and prevented cardiomyocyte apoptosis to a greater extent than aged MSCs, and had improved heart function and cell survival more effectively than aged MSCs four weeks after MI. Thus, MIF rejuvenated aged MSCs by activating autophagy and enhanced their therapeutic efficacy in MI, suggesting a novel MSC-based therapeutic strategy for cardiovascular diseases in the aged population.

## INTRODUCTION

Despite advances in pharmacological therapy and surgical intervention, myocardial infarction (MI) remains the leading cause of morbidity and mortality worldwide. Mesenchymal stem cell (MSC)-based therapy is a novel treatment strategy for MI, and MSCs have the unique advantages of easy isolation, low immunogenicity and multiple lineage potential [1–3]. The therapeutic activity of MSCs in MI has been predominantly attributed to their paracrine effects, rather than their differentiation [2, 4].

Most patients who experience MI are elderly. Autologous MSCs isolated from aged individuals have dramatically lower functional capacities (e.g., ability to survive in the ischemic heart and exert paracrine effects) than those from young individuals, and thus have limited therapeutic efficacy for MI [1, 5–7]. Although the transplantation of allogeneic young MSCs has similar early benefits to the use of autologous MSCs, immune rejection is inevitable and reduces the long-term benefits of allogeneic young MSCs [8]. Therefore, it is greatly important to explore novel strategies of rejuvenating autologous aged MSCs to enhance their benefits for MI treatment. Recent studies have revealed that genetic modification is an effective way to rejuvenate aged MSCs [8, 9].

Macrophage migration inhibitory factor (MIF), an inflammatory cytokine, is widely expressed in various cell types, including MSCs [10]. MIF is involved in multiple signaling pathways, including those that promote cell proliferation and survival [11, 12]. MIF has been reported to prevent cellular senescence [13, 14], and was found to inhibit doxorubicin-induced MSC senescence by activating the phosphoinositide 3-kinase/Akt signaling pathway [15]. Nevertheless, the mechanisms underlying the effects of MIF remain largely unidentified.

Autophagy is a highly conserved process whereby harmful cytoplasmic components are degraded in the lysosomes [16]. Impaired autophagy is associated with aging [17]; indeed, basal autophagy is essential for maintaining cells at a young stage, and deficient autophagy induces cellular senescence [18]. Importantly, autophagy promotes the paracrine effects of MSCs [19]. MIF has been documented to downregulate autophagy [20, 21]. In the current study, we found that MIF rejuvenated aged MSCs by activating autophagy, thus improving the survival of aged MSCs and increasing their therapeutic efficacy in MI.

## RESULTS

### Characterization of young and aged MSCs

We isolated MSCs from the bone marrow of young and aged donors. Then, we used flow cytometry to examine the cell surface marker expression of the young and aged MSCs. Young and aged MSCs expressed surface markers in similar patterns, as they were CD73 (+), CD90 (+), CD105 (+), CD45 (-) and CD34 (-) (Supplementary Figure 1A). We also examined the adipogenic and osteogenic differentiation capacities of the young and aged MSCs. Both types of MSCs could differentiate into adipocytes, as evidenced by Oil Red O staining (Supplementary Figure 1B). Notably, aged MSCs exhibited a higher adipogenic differentiation capacity than young MSCs (Supplementary Figure 1B). Furthermore, aged MSCs had a significantly lower osteogenic differentiation capacity than young MSCs, as evidenced by Alizarin Red staining (Supplementary Figure 1C). Thus, consistent with a previous study [22], aged MSCs exhibited a greater adipogenic differentiation potential and a lower osteogenic differentiation potential than young MSCs.

### Aged MSCs display increased cellular senescence and reduced function

**Figure 1:**
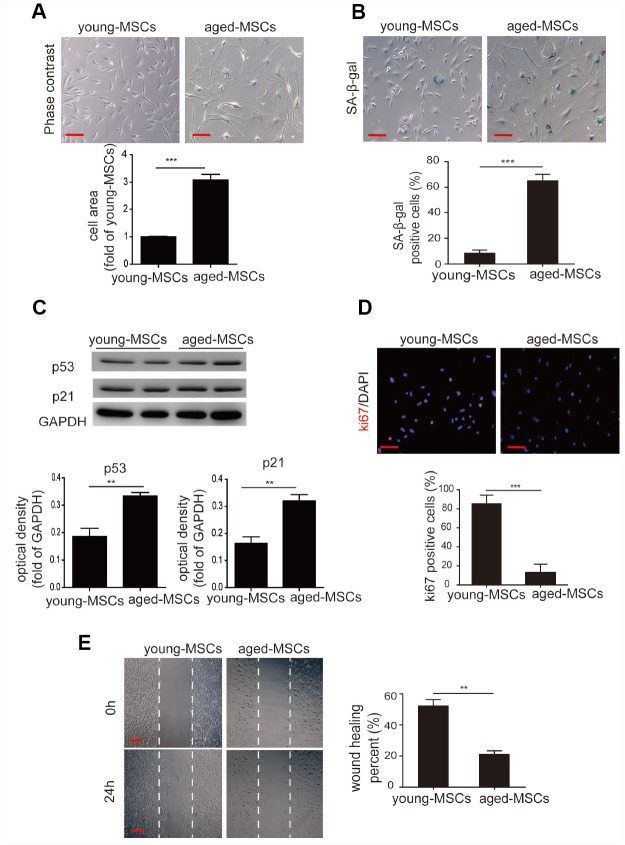
**Aged MSCs display increased cellular senescence.** (**A**) Representative images of young and aged MSCs under light microscopy, and quantitative analysis of the cell size of young and aged MSCs. (**B**) Representative images of SA-β-gal staining, and quantitative analysis of SA-β-gal-positive cells among young and aged MSCs. (**C**) Western blotting and quantitative analysis of p53 and p21 protein expression in young and aged MSCs. (**D**) Representative images of Ki-67 staining and quantitative analysis of Ki-67-positive cells among young and aged MSCs. (**E**) Representative images of the wound healing assay and quantitative analysis of the wound recovery rate in young and aged MSCs. Scale bar=100 μm. Data are expressed as the mean±SEM. n=3. **p<0.01; ***p<0.001.

Given that an altered differentiation potential is a sign of MSC aging, we examined the cellular senescence of young and aged MSCs. We first examined the morphology of the cells, and found that young MSCs were spindle-shaped and fibroblast-like, whereas aged MSCs appeared flattened and were significantly larger than young MSCs (Figure 1A). Next, we performed senescence-associated-β-galactosidase (SA-β-gal) staining to assess cellular senescence. We detected a higher number of SA-β-gal-positive cells among aged MSCs than among young MSCs (Figure 1B). Western blotting also revealed that the senescence-associated markers p21 and p53 were expressed at significantly higher levels in aged MSCs than in young MSCs (Figure 1C).

We then assessed cellular proliferation with Ki-67 staining, and found that the percentage of Ki-67-positive cells was much lower among aged MSCs than among young MSCs (Figure 1D). Using a wound healing assay, we also evaluated the migration abilities of aged and young MSCs. After the cells had been stimulated for 24 hours with 1% serum, the extent of wound closure was markedly lower for aged MSCs than for young MSCs (Figure 1E).

To further evaluate the function of aged and young MSCs, we isolated conditioned medium (CdM) from aged and young MSCs, and co-cultured the CdM with neonatal rat cardiomyocytes under a serum deprivation/hypoxia (SD/H) challenge for 48 hours. MSC-CdM treatment robustly inhibited the SD/H-induced apoptosis of neonatal rat cardiomyocytes, as evidenced by terminal deoxynucleotidyl transferase dUTP nick end labeling (TUNEL) staining (Supplementary Figure 2A). However, the apoptosis of neonatal rat cardiomyocytes was reduced much further by young MSC-CdM than by aged MSC-CdM (Supplementary Figure 2A). MSC-CdM also induced the tube formation of human umbilical vein endothelial cells, but young MSC-CdM stimulated tube formation to a greater extent than aged MSC-CdM (Supplementary Figure 2B). Thus, MSCs isolated from aged individuals exhibited cellular senescence.

### MIF inhibits the cellular senescence of MSCs

**Figure 2:**
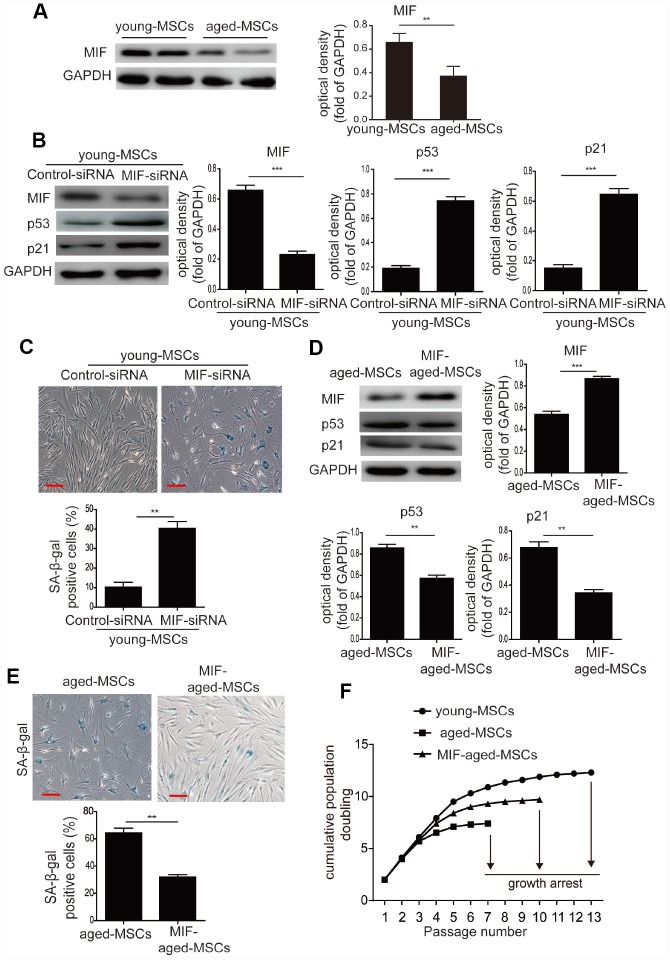
**MIF inhibited the cellular senescence of MSCs.** (**A**) Western blotting and quantitative analysis of MIF protein expression in young and aged MSCs. (**B**) Western blotting and quantitative analysis of MIF, p53 and p21 protein expression in young MSCs treated with control siRNA or MIF-siRNA. (**C**) Representative images of SA-β-gal staining and quantitative analysis of SA-β-gal-positive cells in young MSCs treated with control siRNA or MIF-siRNA. (**D**) Western blotting and quantitative analysis of MIF, p53 and p21 protein expression in aged and MIF-aged MSCs. (**E**) Representative images of SA-β-gal staining and quantitative analysis of SA-β-gal-positive cells in aged and MIF-aged MSCs. (**F**) Cell growth curves showed that overexpression of MIF in aged MSCs increased their growth rate. Scale bar=100 μm. Data are expressed as the mean±SEM. n=3. **p<0.01; ***p<0.001.

To determine whether MIF regulates the senescence of MSCs, we examined the expression of MIF in young and aged MSCs. MIF protein levels were significantly lower in aged MSCs than in young MSCs (Figure 2A), indicating that MIF expression was inversely associated with cellular senescence. We then used small interfering RNA (siRNA) to knock down MIF in young MSCs. MIF-siRNA treatment of young MSCs significantly downregulated MIF expression (Supplementary Figure 3), while it significantly elevated p53 and p21 expression (Figure 2B). Furthermore, MIF-siRNA treatment remarkably increased the number of SA-β-gal-positive cells among young MSCs (Figure 2C).

Next, aged MSCs were transduced with a lentiviral vector containing *MIF* cDNA and green fluorescent protein (*GFP*) (‘MIF-aged MSCs’) or a vector containing *GFP* alone (‘aged MSCs’). GFP fluorescence was observed in both aged and MIF-aged MSCs under a microscope (Supplementary Figure 4A). Most MIF-aged MSCs (>90%) expressed GFP, indicating that MIF was successfully transduced (Supplementary Figure 4B). Furthermore, MIF-aged MSCs expressed CD73, CD90 and CD105, but not CD45 or CD34 (data not shown). While the overexpression of MIF in aged MSCs enhanced MIF expression, it reduced p53 and p21 expression (Figure 2D). Moreover, the percentage of SA-β-gal-positive cells was greatly reduced in MIF-aged MSCs compared with aged MSCs (Figure 2E). We also used serial passaging to examine the growth of young, aged and MIF-aged MSCs. Aged MSCs grew at a lower rate than young MSCs and became arrested at passage 7, whereas young MSCs continued growing until passage 13 (Figure 2F). Overexpression of MIF in aged MSCs increased their growth rate and delayed the arrest of their growth until passage 10 (Figure 2F). These findings suggest that MIF inhibits the cellular senescence of MSCs, and that overexpression of MIF can attenuate the senescence of aged MSCs.

### MIF rejuvenates aged MSCs by promoting autophagy

Autophagy has recently been found to inhibit cellular senescence [23, 24]. Thus, we examined the expression of key autophagy-associated proteins (LC3I/II, Beclin1 and p62) in young and aged MSCs. Beclin1 and LC3I/II levels were much lower and p62 expression was significantly higher in aged MSCs than in young MSCs, suggesting that autophagy was suppressed in aged MSCs (Supplementary Figure 5A).

**Figure 3:**
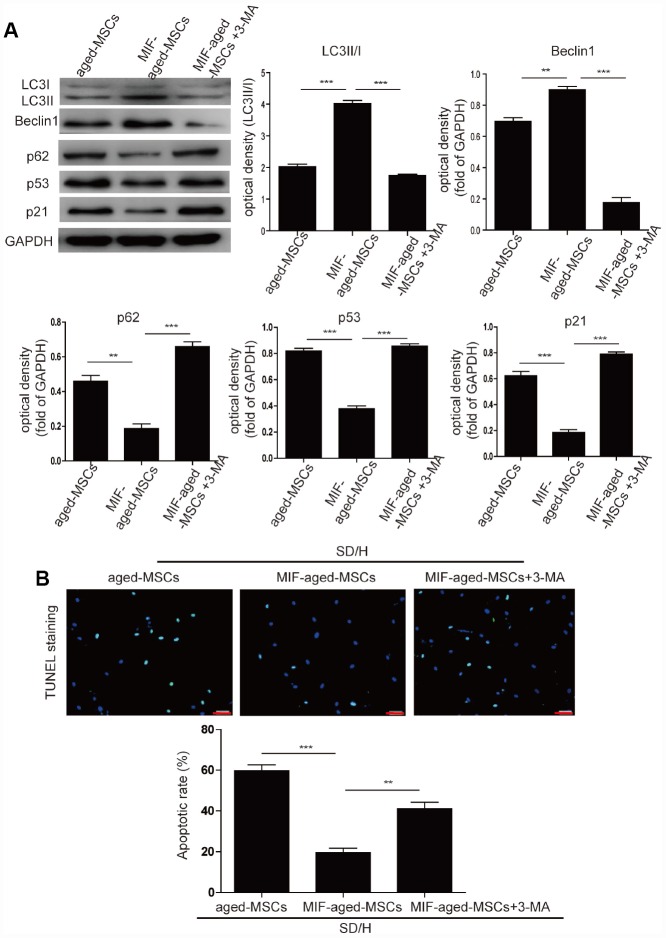
**MIF rejuvenated aged MSCs by promoting autophagy.** (**A**) Western blotting and quantitative analysis of LC3I/II, Beclin1, p62, p53 and p21 protein expression in aged MSCs and MIF-aged MSCs with or without 3-MA treatment. (**B**) Representative images of TUNEL staining and quantitative analysis of the apoptosis of aged MSCs and MIF-aged MSCs with or without 3-MA treatment under an SD/H challenge. Scale bar=100 μm. Data are expressed as the mean±SEM. n=3. **p<0.01; ***p<0.001.

Next, we investigated whether MIF inhibits MSC senescence by activating autophagy. Knockdown of MIF in young MSCs significantly downregulated Beclin1 and LC3I/II and upregulated p62, suggesting that MIF promotes autophagy in MSCs (Supplementary Figure 5B). Overexpression of MIF in aged MSCs significantly induced autophagy, as manifested by the elevated expression of Beclin1 and LC3I/II and the reduced expression of p62 (Figure 3A). Considering these results together with the suppression of p53 and p21 expression in MIF-aged MSCs compared with aged MSCs, we concluded that MIF rejuvenated aged MSCs by activating autophagy. To verify this conclusion, we treated MIF-aged MSCs with the autophagy inhibitor 3-methyladenine (3-MA). Treatment with 3-MA reduced the autophagy and increased the p53 and p21 levels of MIF-aged MSCs (Figure 3A).

We then examined the survival of aged and MIF-aged MSCs under an SD/H challenge. After a 48-hour SD/H challenge, MIF-aged MSCs underwent apoptosis to a remarkably lower extent than aged MSCs (Figure 3B). This protective effect of MIF on aged MSCs was largely reversed by 3-MA treatment (Figure 3B). Furthermore, the tube formation capacity (i.e., tube length) of human umbilical vein endothelial cells was significantly greater in MIF-aged MSC-CdM than in aged MSC-CdM (Supplementary Figure 5C). Treatment with 3-MA partially reversed the enhanced angiogenic potential of MIF-aged MSCs (Supplementary Figure 5C).

To further explore the angiogenic potential of young MSC-CdM, aged MSC-CdM, MIF-aged MSC-CdM and MIF-aged MSC+3-MA-CdM, we used an antibody array to measure the secretion of angiogenic factors from the CdM. The levels of the proangiogenic proteins angiogenin, angiopoietin-2 (ANG-2), epidermal growth factor (EGF) and vascular endothelial growth factor (VEGF) were significantly lower in aged MSC-CdM than in young MSC-CdM (Supplementary Figure 6). Overexpression of MIF in aged MSCs elevated the levels of angiogenin, ANG-2, EGF and VEGF in the CdM, while 3-MA treatment downregulated these factors in MIF-aged MSC-CdM (Supplementary Figure 6). These data suggest that MIF rejuvenated aged MSCs and enhanced their angiogenic potential by activating autophagy.

### Transplantation of MIF-aged MSCs improves cardiac function in a rat model of MI

**Figure 4:**
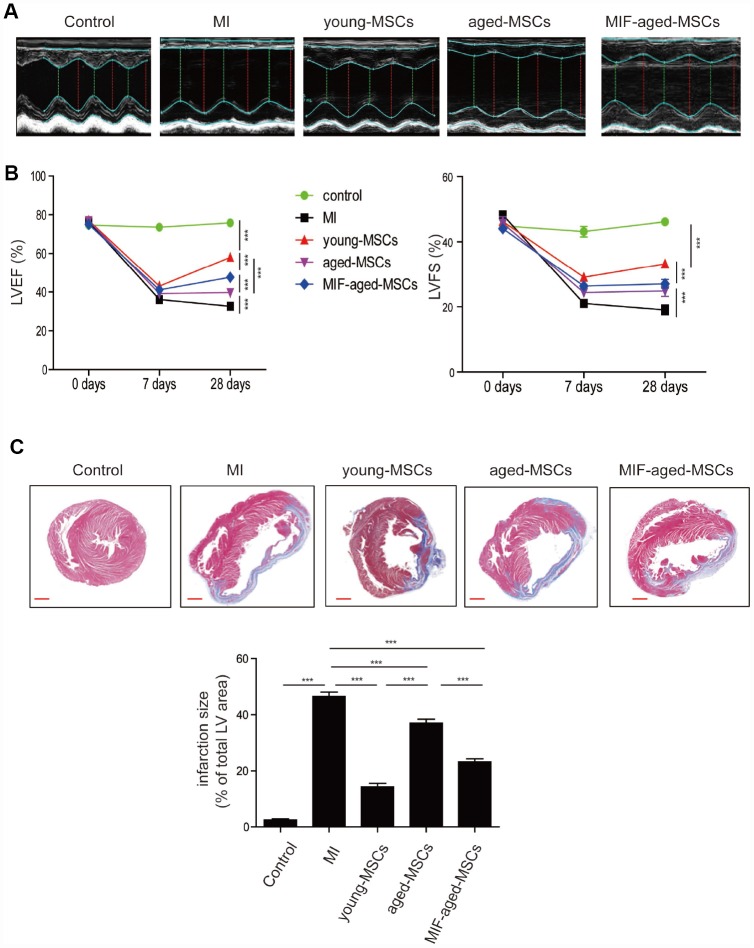
**Transplantation of MIF-aged MSCs improved cardiac function in a rat model of MI.** (**A**) Representative images of M-mode echocardiography captured 28 days after MI in rats from the different groups. (**B**) Heart function, including the LVEF and LVFS, was evaluated at 0, 7 and 28 days in control mice and MI mice with or without MSC transplantation. (**C**) Representative images of Masson's Trichrome staining and quantitative analysis of heart fibrosis in the different experimental groups. Scale bar=2 mm. Data are expressed as the mean±SEM. n=6-7. **p<0.01; ***p<0.001.

Next, we generated a rat model of MI, and examined the effects of MSC injection on cardiac function by performing echocardiography. Representative images of M-mode echocardiography were captured 28 days after MI (Figure 4A). The left ventricular (LV) ejection fraction (LVEF) and fraction shortening (LVFS) were similar among the different groups on day 0, but were much lower in the MI group than in the control group on days 7 and 28 (Figure 4B). In the MSC-treated groups, the LVEF and LVFS gradually increased from days 7 to 28 after MI surgery, while this did not occur in the MI group (Figure 4B). The LVEF and LVFS were lower in the aged MSC group than in the young MSC group, but were much higher in the MIF-aged MSC group than in the aged MSC group 28 days after MI (Figure 4B).

We then performed Masson's Trichrome staining to test for fibrosis in the different groups. Four weeks following MI, the infarct size was significantly lower in the MSC-treated groups than in the MI group (Figure 4C). Notably, the infarct size was much greater in the aged MSC group than in the young MSC group, but was much lower in the MIF-aged MSC group than in the aged MSC group (Figure 4C). Thus, MIF-aged MSCs ameliorated MI-induced cardiac fibrosis more effectively than aged MSCs.

### MIF overexpression enhances cell survival and angiogenesis in the rat heart after MI

**Figure 5:**
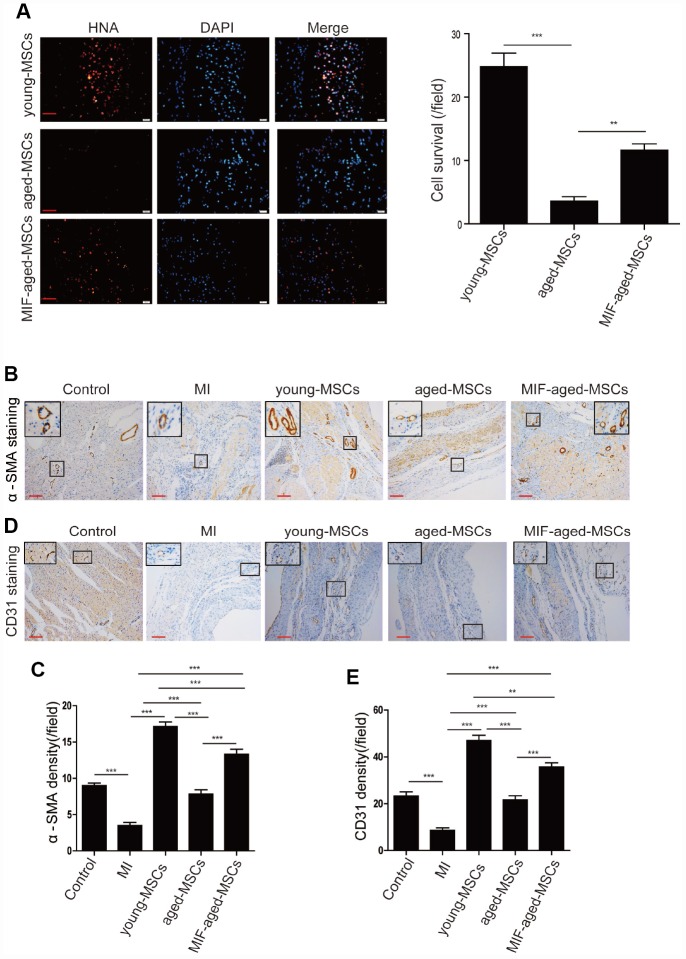
**MIF overexpression enhanced cell survival and angiogenesis in the rat heart after MI**. (**A**) Representative images of HNA staining and quantitative analysis of MSC survival in the heart tissue of rats in the different groups at 28 days. Scale bar=50 μm. (**B**) Representative images of α-SMA staining in the heart tissue of rats from the different groups at 28 days. Scale bar=100 μm. (**C**) Quantitative analysis of the α-SMA density in the heart tissue of rats from the different groups at 28 days. (**D**) Representative images of CD31 staining in the heart tissue of rats from the different groups at 28 days. Scale bar=100 μm. (**E**) Quantitative analysis of the CD31 density in the heart tissue of rats from the different groups at 28 days. Data are expressed as the mean±SEM. n=6-7. **p<0.01; ***p<0.001.

Subsequently, we assessed the survival of MSCs 28 days post-transplantation by performing human nuclear antigen (HNA) staining. As shown in Figure 5A, HNA-positive cells were detected in the hearts of the MSC-treated rats following MI. Although the number of surviving MSCs in the heart was the highest in the young MSC group, it was significantly greater in the MIF-aged MSC group than in the aged MSC group (Figure 5A), suggesting that MIF overexpression improved aged MSC survival following MI.

We then performed alpha smooth muscle actin (α-SMA) staining to detect the blood vessels of the ischemic areas in the different groups by (Figure 5B). The α-SMA density was markedly lower in the MI group than in the control group, but was much greater in the MSC-treated groups than in the MI group (Figure 5C). The α-SMA density was notably higher in the MIF-aged MSC group than in the aged MSC group (Figure 5C).

Lastly, we performed CD31 staining to assess the capillary density of the ischemic areas in the different groups (Figure 5D). The CD31 density was much greater in the MSC-treated groups than in the MI group (Figure 5E). Although the CD31 density was highest in hearts from the young MSC group, it was significantly greater in the MIF-aged MSC group than in the aged MSC group (Figure 5E). These results indicate that MIF overexpression enhanced aged MSC survival and angiogenesis in the rat heart after MI.

## DISCUSSION

The present study had several major findings. First, MIF expression was inversely associated with MSC senescence, so the downregulation of MIF in young MSCs led to cellular senescence. Second, increasing MIF expression rejuvenated aged MSCs by activating autophagy and protected aged MSCs against an SD/H challenge *in vitro*. Third, transplantation of MIF-aged MSCs promoted cell survival and increased angiogenesis more effectively than transplantation of aged MSCs in infarcted rat hearts, thus enhancing cardioprotection after MI.

Although the transplantation of autologous MSCs has had promising results for MI treatment, the therapeutic efficacy of MSCs may be hampered by intrinsic factors such as aging [25, 26]. MSCs isolated from young and aged populations exhibit different biological properties and functions; for instance, aged MSCs have a lower proliferative capacity, differentiation potential and anti-apoptotic capacity than young MSCs [27–29]. A previous study indicated that aged MSCs were much more prone to cellular senescence than young MSCs, and exhibited a senescence-associated secretory phenotype characterized by inflammatory cytokine secretion, which further reduced their therapeutic efficacy [30]. Cellular senescence has also been reported to impair the angiogenic potential of MSCs [31]. In the current study, MSCs isolated from an older population were larger, exhibited greater SA-β-gal activity and displayed lower proliferation and migration capacities than young MSCs. Furthermore, the adipogenic differentiation capacity of aged MSCs was greatly increased, while their osteogenic differentiation capacity was reduced. The angiogenic capacity of aged MSC-CdM was also significantly lower than that of young MSC-CdM. These results imply that the functions of aged MSCs are impaired.

Various strategies have been explored to rejuvenate aged MSCs, including pharmacological pretreatment, hypoxic preconditioning and genetic modification [32–36]. Overexpression of FOXQ1 inhibited the senescence and improved the migration of human umbilical cord MSCs *in vitro* and *in vivo*, thus promoting their therapeutic efficacy in an animal model of Alzheimer's disease [37]. Overexpression of YAP or FOXD1 alleviated the senescence of aged MSCs and attenuated the development of osteoarthritis in mice [38]. Nevertheless, the mechanisms underlying cellular senescence are not fully understood. Given that genetic modifications can rejuvenate aged MSCs, identifying a key regulator of MSC senescence would enable the development of novel strategies to increase the therapeutic efficacy of aged MSCs.

MIF, originally found to be released by activated T cells, is a pleiotropic inflammatory mediator with multiple functions [39]. The activity of MIF as an aging suppressor has been intensively studied. MIF knockout mice developed spontaneous emphysema by six months of age due to the activation of the senescent pathway markers p53/21 and p16. These mice were also more susceptible than wild-type mice to the development of chronic obstructive pulmonary disease, an aging-related condition [40]. Treating aged MSCs with MIF was reported to improve their growth, their survival under a hypoxic challenge and their paracrine effects by activating the CD74/AMPK/FOXO3a signaling pathway, indicating that MIF confers resistance to senescence [41].

In the current study, MIF protein expression was found to be significantly lower in aged MSCs than in young MSCs. The knockdown of MIF with siRNA induced cellular senescence in young MSCs, whereas the overexpression of MIF with a lentiviral expression vector attenuated senescence in aged MSCs, implying that MIF inhibits the senescence of MSCs. In line with previous studies, we observed that the overexpression of MIF in aged MSCs significantly improved their survival under an SD/H challenge. MIF overexpression also increased the cell survival and cardioprotective effects of aged MSCs in the ischemic hearts of rats, as evidenced by the increased cardiac function and reduced scar size in rats treated with MIF-aged MSCs. These results indicate that overexpressing MIF can rejuvenate aged MSCs and restore their functions, although the mechanisms remain to be fully delineated.

Autophagy has been increasingly recognized for its anti-aging effects [42, 43]. Hypoxia pretreatment was found to delay MSC aging by activating autophagy through the hypoxia-inducible factor 1α/AIMP3 signaling pathway [23]. The autophagy inhibitor 3-MA was reported to induce aging in young MSCs, whereas the autophagy activator rapamycin rejuvenated aged MSCs into a young state [22]. Therefore, activating autophagy may be a promising strategy to restore the function of aged MSCs. We found that the level of autophagy was dramatically enhanced in MIF-aged MSCs compared with aged MSCs. Considered along with the reduced senescence of MIF-aged MSCs, these results suggest that MIF overexpression rejuvenated aged MSCs by activating autophagy. As expected, an autophagy inhibitor partially abrogated the senescence-attenuating effects of MIF in aged MSCs.

We also observed that the extent of apoptosis under an SD/H challenge was much lower in MIF-aged MSCs than in aged MSCs. Ample evidence has revealed that the activation of autophagy can protect cells from apoptosis under stress [44, 45]. The increased survival of MIF-aged MSCs under an SD/H challenge was also largely reversed by an autophagy inhibitor. These results indicate that MIF protected MSCs from apoptosis partly by activating autophagy.

Autophagy activation has been reported to enhance the proangiogenic activity of MSCs [46]. We found that proangiogenic proteins including angiogenin, ANG-2, EGF and VEGF were significantly upregulated in MIF-aged MSC-CdM compared with aged MSC-CdM, while these effects were largely abrogated by the autophagy inhibitor 3-MA. These results suggest that MIF increased the expression of proangiogenic factors in aged MSCs by promoting autophagy.

There are some limitations to our study. First, although we observed no teratogenic effects of MIF-overexpressing MSCs in severe combined immunodeficiency mice in the current study (data not shown), genetically modified MSCs need to be carefully evaluated prior to their application in clinical practice. Second, although our results demonstrated that MIF rejuvenated aged MSCs by activating autophagy, the mechanisms for these effects remain to be determined. Third, the rejuvenation of aged MSCs by MIF was not entirely reversed by the autophagy inhibitor 3-MA, indicating that other signaling pathways may also participate in this process. Fourth, we did not evaluate the long-term effects of MIF-aged MSCs on heart function and survival in our rat model of MI.

In summary, our study demonstrated that MIF is a novel factor that can rejuvenate aged MSCs by activating autophagy, thus improving their survival under conditions of SD/H and enhancing their therapeutic efficacy for MI. Therefore, MIF-rejuvenated aged MSCs may provide a cardiac repair strategy for aged hearts following MI.

## MATERIALS AND METHODS

### Cell culture

Young and aged human bone marrow MSCs were isolated from young (n=3, 18-25 years old) and aged donors (n=3, 72-80 years old), respectively, as previously reported [1]. The procedure was approved by the research ethics board of Shanghai East Hospital, and written informed consent was obtained from all donors. MSCs were cultured as described previously [1]. MSCs were used in the current study at passage 3-4. Neonatal rat cardiomyocytes were isolated and cultured as described previously [47].

### Characterization of MSCs and lentiviral transduction

The surface antigen expression of MSCs was determined by flow cytometry. The following antibodies were used: anti-CD29 (Biolegend, 303003), anti-CD45 (Biolegend, 304011), anti-CD73 (Biolegend, 344003), anti-CD90 (Biolegend, 328107) and anti-CD105 (Biolegend, 323205). Aged MSCs were transfected with either a lentivirus containing *MIF* (MIF-aged MSCs) or a control vector (aged MSCs), as previously described [48]. The transfection efficiency was examined by fluorescence microscopy and Western blotting. The capacity of MSCs to differentiate into osteocytes and adipocytes was examined as previously reported [48].

### SA-β-gal staining

The senescence of MSCs from the different groups was determined with an SA-β-gal staining kit (C0602, Beyotime). Briefly, MSCs were plated on a 24-well plate with cover slides. After different treatments, the MSCs were washed with phosphate-buffered saline (PBS) and fixed for 30 minutes. Subsequently, the MSCs were incubated with an SA-β-gal staining solution overnight at 37°C. Finally, the samples were washed and photographed. The percentage of senescent MSCs was calculated as the ratio of blue (positive) MSCs to all MSCs.

### SiRNA transfection

MSCs were transfected with MIF-siRNA (sc-37137, Santa Cruz) or control siRNA (sc-37007, Santa Cruz) by means of a Lipofectamine RNAiMAX Reagent Kit (Invitrogen, 13778030) according to the manufacturer’s protocol. The transfection efficiency was determined by Western blotting 72 hours after transfection.

### Scratch-wound assay

Young and aged MSCs were seeded into a six-well plate and cultured. Scratches of the same width were made on the bottom of each well with a 1-mL pipette tip. The wells were washed with PBS to remove cell debris, and the MSCs were then cultured in serum-free medium. After 24 hours of culture, the migration of MSCs into the “wounded” area was photographed and evaluated.

### TUNEL assay

The apoptosis of MSCs under an SD/H challenge was evaluated by TUNEL staining (11767291910, Roche). Briefly, MSCs from the different groups were seeded into a 24-well plate with cover slides, and were exposed to SD/H (1% O_2_, 5% CO_2_ and 94% N_2_) for 48 hours. TUNEL staining was performed according to the manufacturer’s protocol. Finally, the samples were mounted with 4’,6-diamidino-2-phenylindole (DAPI) and photographed. The apoptotic rate of MSCs was determined from six different view fields for each sample in three independent experiments.

### Western blotting

Proteins were extracted from MSCs, and the protein concentration was measured with a bicinchoninic acid assay kit (Thermo, 231227). After separation by sodium dodecyl sulfate polyacrylamide gel electrophoresis, the proteins were transferred onto polyvinylidene difluoride membranes. The membranes were blocked with 5% skim milk in Tris-buffered saline-Tween, and then were incubated overnight at 4°C with the following antibodies: anti-p21 (ab109199, Abcam), anti-p53 (ab26, Abcam), anti-LC3I/II (#12741, CST), anti-Beclin1 (#3738, CST), anti-p62 (#5114, CST) and anti-GAPDH (#2118, CST). After being washed with Tris-buffered saline-Tween three times, the membranes were incubated with secondary antibodies (1:5000, CST) at 37°C for one hour and then exposed to radiography film in a dark room.

### CdM collection and angiogenic cytokine analysis

CdM was prepared from young, aged and MIF-aged MSCs as previously reported [47]. Briefly, 5×10^6^ young, aged and MIF-aged MSCs were plated on 15-cm culture dishes and cultured until they reached 70-80% confluence. The cells were washed three times with PBS, and then 15 mL of serum- and antibiotic-free Dulbecco’s modified Eagle’s medium was added. Twenty-four hours later, the supernatants of the young, aged and MIF-aged MSCs were collected and centrifuged (4,000 x *g* for 30 minutes at 4°C) in ultrafiltration conical tubes (Amicon Ultra-15 with membranes selective for 3 kDa) to concentrate the CdM. Finally, the concentration of the CdM was measured. The angiogenic cytokines in the MSC-CdM were assessed with a RayBio® Cytokine Antibody Array (QAH-ANG-1) as previously described [49]. The cytokines were quantified with a Quantibody Q-Analyzer.

### Neonatal rat cardiomyocyte and MSC-CdM culture

To examine the protective effects of MSC-CdM, we co-cultured neonatal rat cardiomyocytes with MSC-CdM and then challenged the cells with SD/H. Forty-eight hours later, the apoptosis of the neonatal rat cardiomyocytes was examined by TUNEL staining.

### Tube formation assay with MSC-CdM

The angiogenetic capacity of CdM from MSCs was evaluated with an In Vitro Angiogenesis Assay Kit (Chemicon, Temecula, CA, USA) according to the manufacturer’s protocol. Briefly, 10,000 human umbilical vein endothelial cells were cultured on a Matrigel-coated 96-well plate. The cells were treated with CdM from MSCs for six hours at 37°C. Finally, tube formation was randomly photographed, and the lengths of the tubes were analyzed.

### MI model and echocardiography

All the animal studies were approved by the Committee on the Use of Live Animals in Teaching and Research at Nanjing Medical University. An acute MI model involving ligation of the left anterior descending coronary artery was established in rats. Briefly, adult Sprague-Dawley rats weighing 220-250 g were anesthetized with intraperitoneal ketamine (100 mg/kg) and xylazine (10 mg/kg) and connected to a ventilator with an orotracheal tube. After fixation, a left thoracotomy was performed between the third and fourth intercostal spaces under sterile conditions. The heart was exposed and the left anterior descending coronary artery was quickly ligated with a 6-0 silk suture. After ligation, the rats were randomly allocated to receive one of the following treatments via intramuscular injections at four sites around the border zone of the infarcted heart: 1) PBS (MI group, n=10); 2) 1×10^6^ young MSCs (young MSC group, n=10); 3) 1×10^6^ aged MSCs (aged MSC group, n=10) or 1×10^6^ MIF-aged MSCs (MIF-aged MSC group, n=10). All MSCs were suspended in 100 μL of PBS. Another group of rats (n=8) underwent thoracotomy without left anterior descending coronary artery ligation, and served as the control group. Cardiac function was examined by echocardiography (Ultramark 9; Soma Technology, Bloomfield, CT, USA) four weeks after the MSC injection. The LVEF and LVFS were calculated.

### Masson's Trichrome staining

The hearts of the rats were harvested, fixed, embedded in paraffin and cut into 5-μm sections. The fibrosis of the hearts from each experimental group was determined with a Masson’s Trichrome staining kit according to the manufacturer’s protocol (HT15, Sigma, St. Louis, MO, USA). The percent infarct size was calculated as (fibrosis area/total LV area)×100%.

### Immunofluorescent staining

Immunofluorescent staining was performed as in our previous study [50]. Heart sections were rehydrated and treated with 3% H_2_O_2_ for 30 minutes. After being washed with PBS three times, the sections were blocked with 5% bovine serum albumin for 30 minutes and then incubated with the following primary antibodies at 4°C overnight: anti-HNA (ab191181, Abcam), anti-α-SMA (SC-53142, Santa Cruz) and anti-CD31 (SC-31054, Santa Cruz). For the negative control, samples were incubated with PBS instead of the primary antibody. Subsequently, the sections were incubated with the appropriate secondary antibodies at room temperature for one hour. Finally, the sections were mounted with DAPI.

### Statistical analysis

Data are expressed as the mean ± standard error of the mean (SEM). Statistical analyses were performed with Prism 5.04 Software (GraphPad Software for Windows, San Diego, CA, USA). An unpaired Student’s *t*-test was used to compare two groups, while one-way analysis of variance followed by the Bonferroni test was used to compare more than two groups. A value of *p*<0.05 was considered statistically significant.

## Supplementary Material

Supplementary Figures

## References

[1] Liang X, Ding Y, Lin F, Zhang Y, Zhou X, Meng Q, Lu X, Jiang G, Zhu H, Chen Y, Lian Q, Fan H, Liu Z. Overexpression of ERBB4 rejuvenates aged mesenchymal stem cells and enhances angiogenesis via PI3K/AKT and MAPK/ERK pathways. FASEB J. 2019; 33:4559–70. 10.1096/fj.201801690R 30566395

[2] Zhang Y, Chiu S, Liang X, Chai YH, Qin Y, Wang J, Li X, Qiu B, Tergaonkar V, Tse HF, Lian Q. Absence of NUCKS augments paracrine effects of mesenchymal stem cells-mediated cardiac protection. Exp Cell Res. 2017; 356:74–84. 10.1016/j.yexcr.2017.04.012 28412246

[3] Luger D, Lipinski MJ, Westman PC, Glover DK, Dimastromatteo J, Frias JC, Albelda MT, Sikora S, Kharazi A, Vertelov G, Waksman R, Epstein SE. Intravenously Delivered Mesenchymal Stem Cells: Systemic Anti-Inflammatory Effects Improve Left Ventricular Dysfunction in Acute Myocardial Infarction and Ischemic Cardiomyopathy. Circ Res. 2017; 120:1598–613. 10.1161/CIRCRESAHA.117.310599 28232595

[4] Liao S, Zhang Y, Ting S, Zhen Z, Luo F, Zhu Z, Jiang Y, Sun S, Lai WH, Lian Q, Tse HF. Potent immunomodulation and angiogenic effects of mesenchymal stem cells versus cardiomyocytes derived from pluripotent stem cells for treatment of heart failure. Stem Cell Res Ther. 2019; 10:78. 10.1186/s13287-019-1183-3 30845990 PMC6407247

[5] Golpanian S, El-Khorazaty J, Mendizabal A, DiFede DL, Suncion VY, Karantalis V, Fishman JE, Ghersin E, Balkan W, Hare JM. Effect of aging on human mesenchymal stem cell therapy in ischemic cardiomyopathy patients. J Am Coll Cardiol. 2015; 65:125–32. 10.1016/j.jacc.2014.10.040 25593053 PMC4405121

[6] Liu X, Chen H, Zhu W, Chen H, Hu X, Jiang Z, Xu Y, Zhou Y, Wang K, Wang L, Chen P, Hu H, Wang C, et al. Transplantation of SIRT1-engineered aged mesenchymal stem cells improves cardiac function in a rat myocardial infarction model. J Heart Lung Transplant. 2014; 33:1083–92. 10.1016/j.healun.2014.05.008 25034794

[7] Dong J, Zhang Z, Huang H, Mo P, Cheng C, Liu J, Huang W, Tian C, Zhang C, Li J. miR-10a rejuvenates aged human mesenchymal stem cells and improves heart function after myocardial infarction through KLF4. Stem Cell Res Ther. 2018; 9:151. 10.1186/s13287-018-0895-0 29848383 PMC5977543

[8] Song HF, He S, Li SH, Yin WJ, Wu J, Guo J, Shao ZB, Zhai XY, Gong H, Lu L, Wei F, Weisel RD, Xie J, Li RK. Aged Human Multipotent Mesenchymal Stromal Cells Can Be Rejuvenated by Neuron-Derived Neurotrophic Factor and Improve Heart Function After Injury. JACC Basic Transl Sci. 2017; 2:702–16. 10.1016/j.jacbts.2017.07.014 30062183 PMC6059002

[9] Yang M, Wen T, Chen H, Deng J, Yang C, Zhang Z. Knockdown of insulin-like growth factor 1 exerts a protective effect on hypoxic injury of aged BM-MSCs: role of autophagy. Stem Cell Res Ther. 2018; 9:284. 10.1186/s13287-018-1028-5 30359321 PMC6202872

[10] Miller EJ, Li J, Leng L, McDonald C, Atsumi T, Bucala R, Young LH. Macrophage migration inhibitory factor stimulates AMP-activated protein kinase in the ischaemic heart. Nature. 2008; 451:578–82. 10.1038/nature06504 18235500

[11] Soppert J, Kraemer S, Beckers C, Averdunk L, Möllmann J, Denecke B, Goetzenich A, Marx G, Bernhagen J, Stoppe C. Soluble CD74 Reroutes MIF/CXCR4/AKT-Mediated Survival of Cardiac Myofibroblasts to Necroptosis. J Am Heart Assoc. 2018; 7:e009384. 10.1161/JAHA.118.009384 30371153 PMC6201423

[12] De R, Sarkar S, Mazumder S, Debsharma S, Siddiqui AA, Saha SJ, Banerjee C, Nag S, Saha D, Pramanik S, Bandyopadhyay U. Macrophage migration inhibitory factor regulates mitochondrial dynamics and cell growth of human cancer cell lines through CD74-NF-κB signaling. J Biol Chem. 2018; 293:19740–60. 10.1074/jbc.RA118.003935 30366984 PMC6314129

[13] Palumbo S, Tsai TL, Li WJ. Macrophage migration inhibitory factor regulates AKT signaling in hypoxic culture to modulate senescence of human mesenchymal stem cells. Stem Cells Dev. 2014; 23:852–65. 10.1089/scd.2013.0294 24274936

[14] Hu Y, Xia W, Hou M. Macrophage migration inhibitory factor serves a pivotal role in the regulation of radiation-induced cardiac senescencethrough rebalancing the microRNA-34a/sirtuin 1 signaling pathway. Int J Mol Med. 2018; 42:2849–58. 10.3892/ijmm.2018.3838 30226567

[15] Xia W, Hou M. Macrophage migration inhibitory factor rescues mesenchymal stem cells from doxorubicin-induced senescence though the PI3K-Akt signaling pathway. Int J Mol Med. 2018; 41:1127–37. 10.3892/ijmm.2017.3282 29207187

[16] Chen X, He Y, Lu F. Autophagy in Stem Cell Biology: A Perspective on Stem Cell Self-Renewal and Differentiation. Stem Cells Int. 2018; 2018:9131397. 10.1155/2018/9131397 29765428 PMC5896318

[17] Revuelta M, Matheu A. Autophagy in stem cell aging. Aging Cell. 2017; 16:912–15. 10.1111/acel.12655 28782921 PMC5595672

[18] García-Prat L, Martínez-Vicente M, Perdiguero E, Ortet L, Rodríguez-Ubreva J, Rebollo E, Ruiz-Bonilla V, Gutarra S, Ballestar E, Serrano AL, Sandri M, Muñoz-Cánoves P. Autophagy maintains stemness by preventing senescence. Nature. 2016; 529:37–42. 10.1038/nature16187 26738589

[19] An Y, Liu WJ, Xue P, Ma Y, Zhang LQ, Zhu B, Qi M, Li LY, Zhang YJ, Wang QT, Jin Y. Autophagy promotes MSC-mediated vascularization in cutaneous wound healing via regulation of VEGF secretion. Cell Death Dis. 2018; 9:58. 10.1038/s41419-017-0082-8 29352190 PMC5833357

[20] Xu X, Pang J, Chen Y, Bucala R, Zhang Y, Ren J. Macrophage Migration Inhibitory Factor (MIF) Deficiency Exacerbates Aging-Induced Cardiac Remodeling and Dysfunction Despite Improved Inflammation: Role of Autophagy Regulation. Sci Rep. 2016; 6:22488. 10.1038/srep22488 26940544 PMC4778027

[21] Liu Y, Zhao L, Ju Y, Li W, Zhang M, Jiao Y, Zhang J, Wang S, Wang Y, Zhao M, Zhang B, Zhao Y. A novel androstenedione derivative induces ROS-mediated autophagy and attenuates drug resistance in osteosarcoma by inhibiting macrophage migration inhibitory factor (MIF). Cell Death Dis. 2014; 5:e1361. 10.1038/cddis.2014.300 25101674 PMC4454296

[22] Ma Y, Qi M, An Y, Zhang L, Yang R, Doro DH, Liu W, Jin Y. Autophagy controls mesenchymal stem cell properties and senescence during bone aging. Aging Cell. 2018; 17:e12709. 10.1111/acel.12709 29210174 PMC5770781

[23] Kim C, Park JM, Song Y, Kim S, Moon J. HIF1α-mediated AIMP3 suppression delays stem cell aging via the induction of autophagy. Aging Cell. 2019; 18:e12909. 10.1111/acel.12909 30706629 PMC6413650

[24] Zhang M, Du Y, Lu R, Shu Y, Zhao W, Li Z, Zhang Y, Liu R, Yang T, Luo S, Gao M, Zhang Y, Zhang G, et al. Cholesterol Retards Senescence in Bone Marrow Mesenchymal Stem Cells by Modulating Autophagy and ROS/p53/p21^Cip1/Waf1^ Pathway. Oxid Med Cell Longev. 2016; 2016:7524308. 10.1155/2016/7524308 27703600 PMC5040816

[25] Zhai XY, Yan P, Zhang J, Song HF, Yin WJ, Gong H, Li H, Wu J, Xie J, Li RK. Knockdown of SIRT6 Enables Human Bone Marrow Mesenchymal Stem Cell Senescence. Rejuvenation Res. 2016; 19:373–84. 10.1089/rej.2015.1770 26654351

[26] Ji J, Fu T, Dong C, Zhu W, Yang J, Kong X, Zhang Z, Bao Y, Zhao R, Ge X, Sha X, Lu Z, Li J, Gu Z. Targeting HMGB1 by ethyl pyruvate ameliorates systemic lupus erythematosus and reverses the senescent phenotype of bone marrow-mesenchymal stem cells. Aging (Albany NY). 2019; 11:4338–53. 10.18632/aging.102052 31303606 PMC6660056

[27] Zhou S, Greenberger JS, Epperly MW, Goff JP, Adler C, Leboff MS, Glowacki J. Age-related intrinsic changes in human bone-marrow-derived mesenchymal stem cells and their differentiation to osteoblasts. Aging Cell. 2008; 7:335–43. 10.1111/j.1474-9726.2008.00377.x 18248663 PMC2398731

[28] Fan M, Chen W, Liu W, Du GQ, Jiang SL, Tian WC, Sun L, Li RK, Tian H. The effect of age on the efficacy of human mesenchymal stem cell transplantation after a myocardial infarction. Rejuvenation Res. 2010; 13:429–38. 10.1089/rej.2009.0986 20583954

[29] Liu M, Lei H, Dong P, Fu X, Yang Z, Yang Y, Ma J, Liu X, Cao Y, Xiao R. Adipose-Derived Mesenchymal Stem Cells from the Elderly Exhibit Decreased Migration and Differentiation Abilities with Senescent Properties. Cell Transplant. 2017; 26:1505–19. 10.1177/0963689717721221 29113467 PMC5680952

[30] Lunyak VV, Amaro-Ortiz A, Gaur M. Mesenchymal Stem Cells Secretory Responses: Senescence Messaging Secretome and Immunomodulation Perspective. Front Genet. 2017; 8:220. 10.3389/fgene.2017.00220 29312442 PMC5742268

[31] De Barros S, Dehez S, Arnaud E, Barreau C, Cazavet A, Perez G, Galinier A, Casteilla L, Planat-Bénard V. Aging-related decrease of human ASC angiogenic potential is reversed by hypoxia preconditioning through ROS production. Mol Ther. 2013; 21:399–408. 10.1038/mt.2012.213 23070114 PMC3594015

[32] Chang YM, Asokan Shibu M, Tsai CT, Tsai CC, Lin SL, Chang CC, Viswanadha VP, Chen RJ, Chang JH, Huang CY. Alpinate Oxyphyllae extracts enhance the longevity and homing of mesenchymal stem cells and augment their protection against senescence in H9c2 cells. J Cell Physiol. 2019; 234:12042–50. 10.1002/jcp.27867 30515824

[33] Waseem M, Khan I, Iqbal H, Eijaz S, Usman S, Ahmed N, Alam G, Salim A. Hypoxic Preconditioning Improves the Therapeutic Potential of Aging Bone Marrow Mesenchymal Stem Cells in Streptozotocin-Induced Type-1 Diabetic Mice. Cell Reprogram. 2016; 18:344–55. 10.1089/cell.2016.0002 27500307

[34] Ren X, Hu B, Song M, Ding Z, Dang Y, Liu Z, Zhang W, Ji Q, Ren R, Ding J, Chan P, Jiang C, Ye K, Qu J, Tang F, Liu GH. Maintenance of Nucleolar Homeostasis by CBX4 Alleviates Senescence and Osteoarthritis. Cell Rep. 2019; 26:3643–3656.e7. 10.1016/j.celrep.2019.02.088 30917318

[35] Chen G, Zhang Y, Yu S, Sun W, Miao D. Bmi1 Overexpression in Mesenchymal Stem Cells Exerts Antiaging and Antiosteoporosis Effects by Inactivating p16/p19 Signaling and Inhibiting Oxidative Stress. Stem Cells. 2019; 37:1200–11. 10.1002/stem.3007 30895687 PMC6851636

[36] Fang J, Yan Y, Teng X, Wen X, Li N, Peng S, Liu W, Donadeu FX, Zhao S, Hua J. Melatonin prevents senescence of canine adipose-derived mesenchymal stem cells through activating NRF2 and inhibiting ER stress. Aging (Albany NY). 2018; 10:2954–72. 10.18632/aging.101602 30362962 PMC6224246

[37] Zhang T, Wang P, Liu Y, Zhou J, Shi Z, Cheng K, Huang T, Wang X, Yang GL, Yang B, Ma S, Guan F. Overexpression of FOXQ1 enhances anti-senescence and migration effects of human umbilical cord mesenchymal stem cells in vitro and in vivo. Cell Tissue Res. 2018; 373:379–93. 10.1007/s00441-018-2815-0 29500491

[38] Fu L, Hu Y, Song M, Liu Z, Zhang W, Yu FX, Wu J, Wang S, Izpisua Belmonte JC, Chan P, Qu J, Tang F, Liu GH. Up-regulation of FOXD1 by YAP alleviates senescence and osteoarthritis. PLoS Biol. 2019; 17:e3000201. 10.1371/journal.pbio.3000201 30933975 PMC6459557

[39] Zheng L, Gao J, Jin K, Chen Z, Yu W, Zhu K, Huang W, Liu F, Mei L, Lou C, He D. Macrophage migration inhibitory factor (MIF) inhibitor 4-IPP suppresses osteoclast formation and promotes osteoblast differentiation through the inhibition of the NF-κB signaling pathway. FASEB J. 2019; 33:7667–83. 10.1096/fj.201802364RR 30893559

[40] Sauler M, Leng L, Trentalange M, Haslip M, Shan P, Piecychna M, Zhang Y, Andrews N, Mannam P, Allore H, Fried T, Bucala R, Lee PJ. Macrophage migration inhibitory factor deficiency in chronic obstructive pulmonary disease. Am J Physiol Lung Cell Mol Physiol. 2014; 306:L487–96. 10.1152/ajplung.00284.2013 24441872 PMC3949087

[41] Xia W, Zhang F, Xie C, Jiang M, Hou M. Macrophage migration inhibitory factor confers resistance to senescence through CD74-dependent AMPK-FOXO3a signaling in mesenchymal stem cells. Stem Cell Res Ther. 2015; 6:82. 10.1186/s13287-015-0076-3 25896286 PMC4453287

[42] Nakamura S, Oba M, Suzuki M, Takahashi A, Yamamuro T, Fujiwara M, Ikenaka K, Minami S, Tabata N, Yamamoto K, Kubo S, Tokumura A, Akamatsu K, et al. Suppression of autophagic activity by Rubicon is a signature of aging. Nat Commun. 2019; 10:847. 10.1038/s41467-019-08729-6 30783089 PMC6381146

[43] Andrique C, Morardet L, Linares LK, Cissé MY, Merle C, Chibon F, Provot S, Haÿ E, Ea HK, Cohen-Solal M, Modrowski D. Calpain-6 controls the fate of sarcoma stem cells by promoting autophagy and preventing senescence. JCI Insight. 2018; 3:121225. 10.1172/jci.insight.121225 30185659 PMC6171816

[44] Li X, Xie X, Yu Z, Chen Y, Qu G, Yu H, Luo B, Lei Y, Li Y. Bone marrow mesenchymal stem cells-derived conditioned medium protects cardiomyocytes from hypoxia/reoxygenation-induced injury through Notch2/mTOR/autophagy signaling. J Cell Physiol. 2019; 234:18906–16. 10.1002/jcp.28530 30953350

[45] Zou L, Ma X, Lin S, Wu B, Chen Y, Peng C. Bone marrow mesenchymal stem cell-derived exosomes protect against myocardial infarction by promoting autophagy. Exp Ther Med. 2019; 18:2574–82. 10.3892/etm.2019.7874 31555366 PMC6755377

[46] Lee SG, Joe YA. Autophagy mediates enhancement of proangiogenic activity by hypoxia in mesenchymal stromal/stem cells. Biochem Biophys Res Commun. 2018; 501:941–47. 10.1016/j.bbrc.2018.05.086 29772235

[47] Zhang Y, Liang X, Liao S, Wang W, Wang J, Li X, Ding Y, Liang Y, Gao F, Yang M, Fu Q, Xu A, Chai YH, et al. Potent Paracrine Effects of human induced Pluripotent Stem Cell-derived Mesenchymal Stem Cells Attenuate Doxorubicin-induced Cardiomyopathy. Sci Rep. 2015; 5:11235. 10.1038/srep11235 26057572 PMC4460911

[48] Liang X, Ding Y, Zhang Y, Chai YH, He J, Chiu SM, Gao F, Tse HF, Lian Q. Activation of NRG1-ERBB4 signaling potentiates mesenchymal stem cell-mediated myocardial repairs following myocardial infarction. Cell Death Dis. 2015; 6:e1765. 10.1038/cddis.2015.91 25996292 PMC4669719

[49] Zhang Y, Liao S, Yang M, Liang X, Poon MW, Wong CY, Wang J, Zhou Z, Cheong SK, Lee CN, Tse HF, Lian Q. Improved cell survival and paracrine capacity of human embryonic stem cell-derived mesenchymal stem cells promote therapeutic potential for pulmonary arterial hypertension. Cell Transplant. 2012; 21:2225–39. 10.3727/096368912X653020 22776744

[50] Zhang Y, Chiu S, Liang X, Gao F, Zhang Z, Liao S, Liang Y, Chai YH, Low DJ, Tse HF, Tergaonkar V, Lian Q. Rap1-mediated nuclear factor-kappaB (NF-κB) activity regulates the paracrine capacity of mesenchymal stem cells in heart repair following infarction. Cell Death Discov. 2015; 1:15007. 10.1038/cddiscovery.2015.7 27551443 PMC4981000

