# Supplementary material for: Macrophage migration inhibitory factor rejuvenates aged human mesenchymal stem cells and improves myocardial repair
Source: Aging (Albany NY). 2019 Dec 27;11(24):12641–60. doi: 10.18632/aging.102592 (PMC6949107; doi:10.18632/aging.102592)
Supplement: Supplementary Figures [file aging-11-102592-s001..pdf]

## SUPPLEMENTARY FIGURES

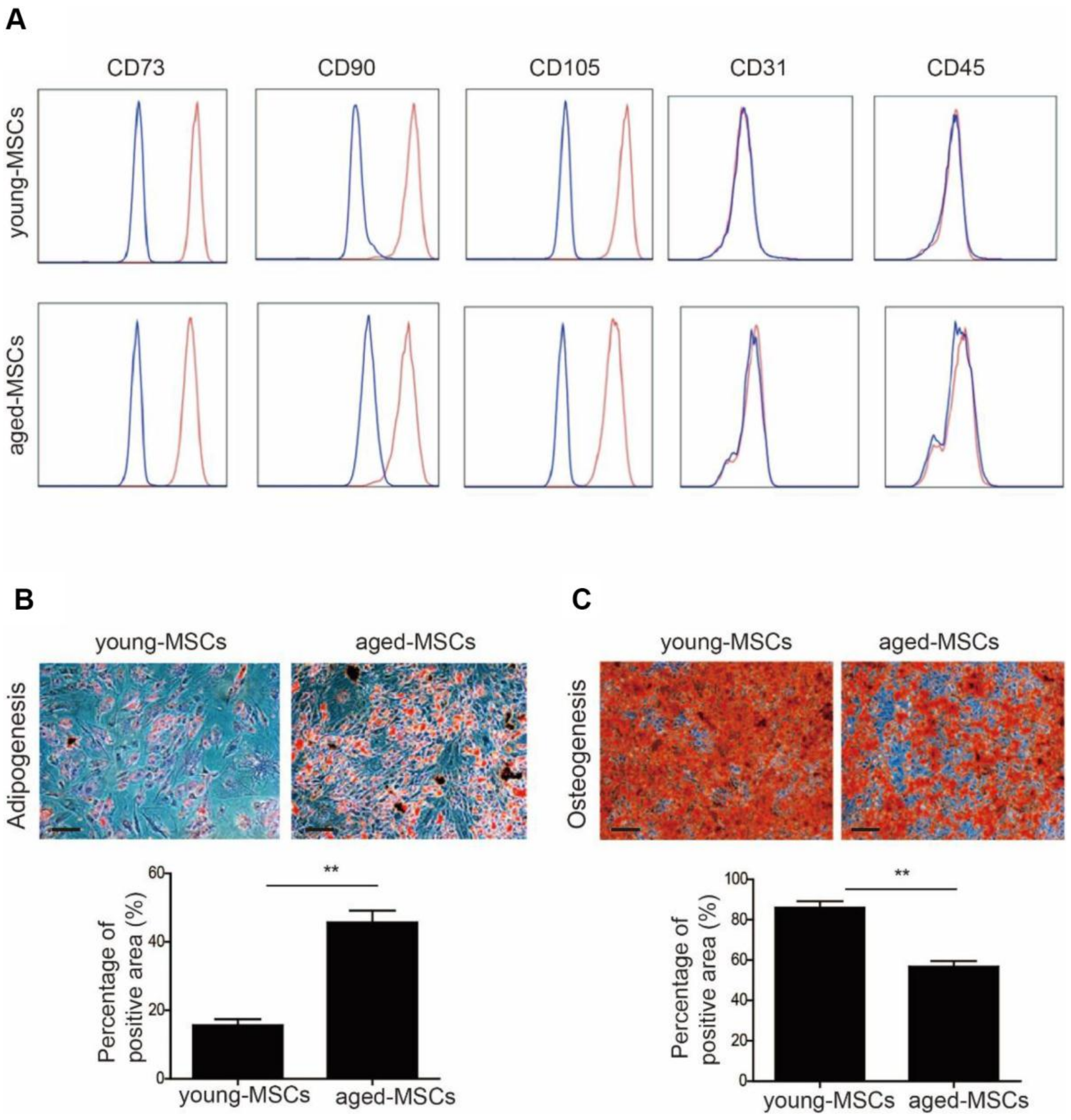


**Supplementary Figure 1. Characterization of young-MSCs and aged-MSCs.** (**A**) Surface marker profiling determined by flow cytometry in young-MSCs and aged-MSCs; that is, negative for CD31and CD45; positive for CD73, CD90 and CD105. (**B**) Oil red staining for adipogenesis and quantification of adipogenic efficiency in young-MSCs and aged-MSCs. (**C**) Alizarin red staining for osteogenesis and quantification of osteogenic efficiency in young-MSCs and aged-MSCs. Scale bar=100μm. Data are expressed as mean±SEM. n=3. ****p<0.01.*

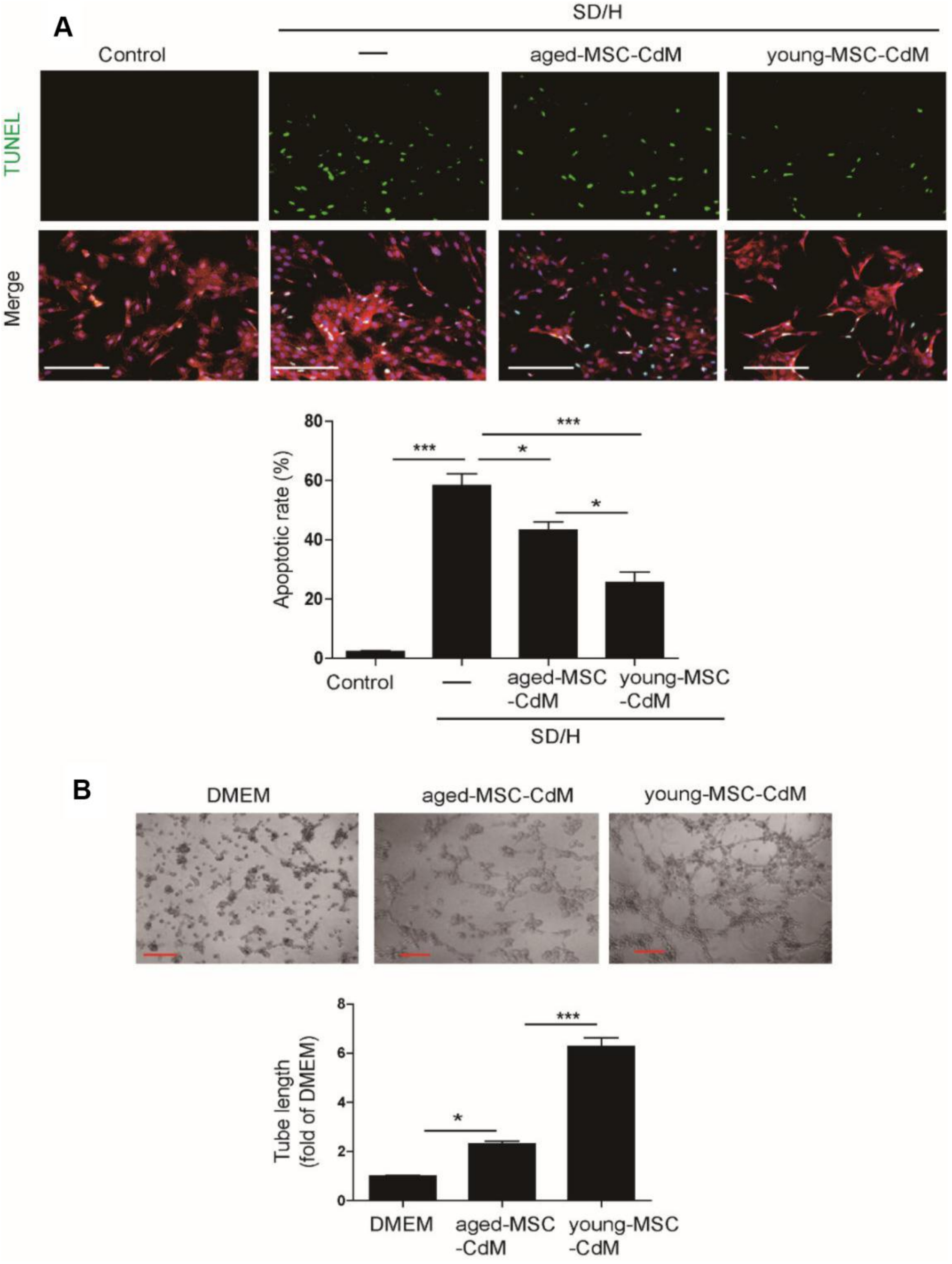


**Supplementary Figure 2. Aged-MSCs exhibited decreased paracrine effects.** (**A**) Representative images of TUNEL staining and quantitative analysis of the apoptotic rate of NCMs co-cultured with DMEM, aged-MSC-CdM or young-MSC-CdM under SD/H challenge. (**B**) Representative images of tube formation and analysis of tube length in HUVECs treated with DMEM, aged-MSC-CdM or young-MSC-CdM. Scale bar=200μm. Data are expressed as the mean±SEM. n=3. **p<0.05; ***p<0.001.*

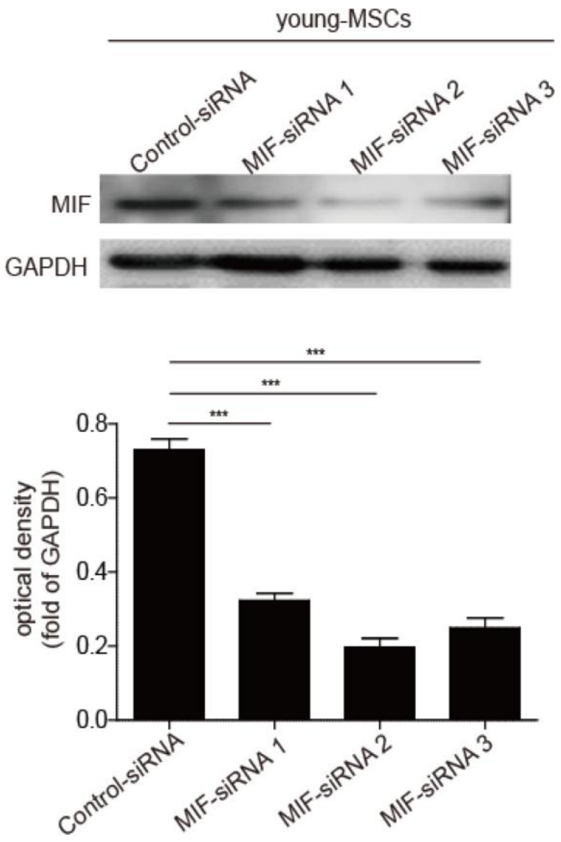


**Supplementary Figure 3. MIF-siRNA treatment significantly downregulated MIF expression in young-MSCs. Data are expressed as the mean±SEM. n=3.** ****p<0.001*.

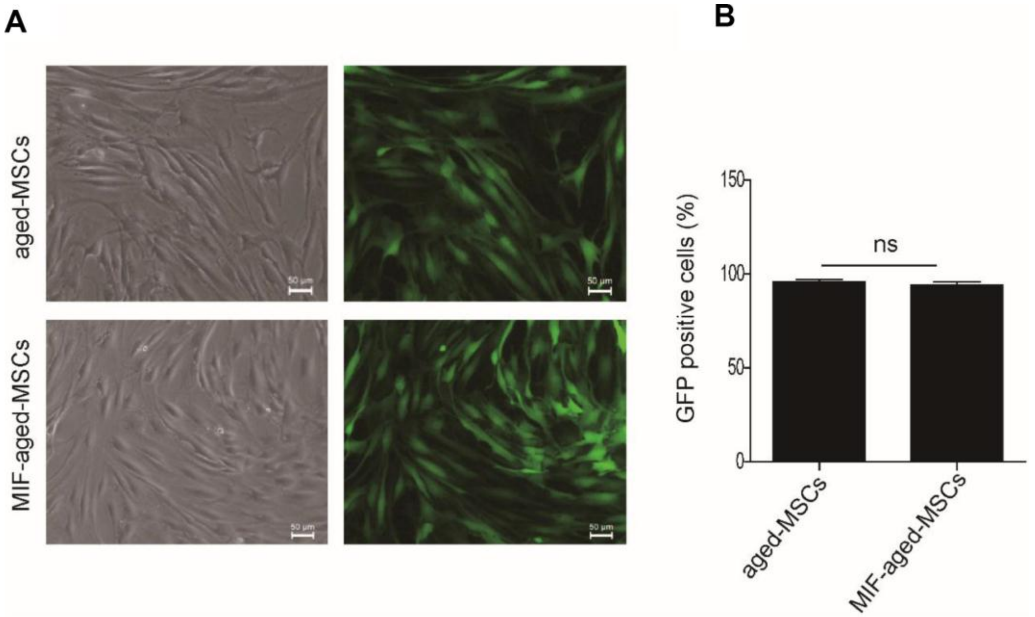


**Supplementary Figure 4. Lentiviral transduction of aged-MSC.** (**A**) Representative images of aged-MSC transduced with lentiviral GFP or lentiviral MIF-GFP under microscope or fluorescence microscope. (**B**) Quantitative analysis of GFP positive aged-MSCs and MIF-aged-MSCs using flow cytometry. Scale bar=50μm. Data are expressed as the mean±SEM. n=3. ns, not significant.

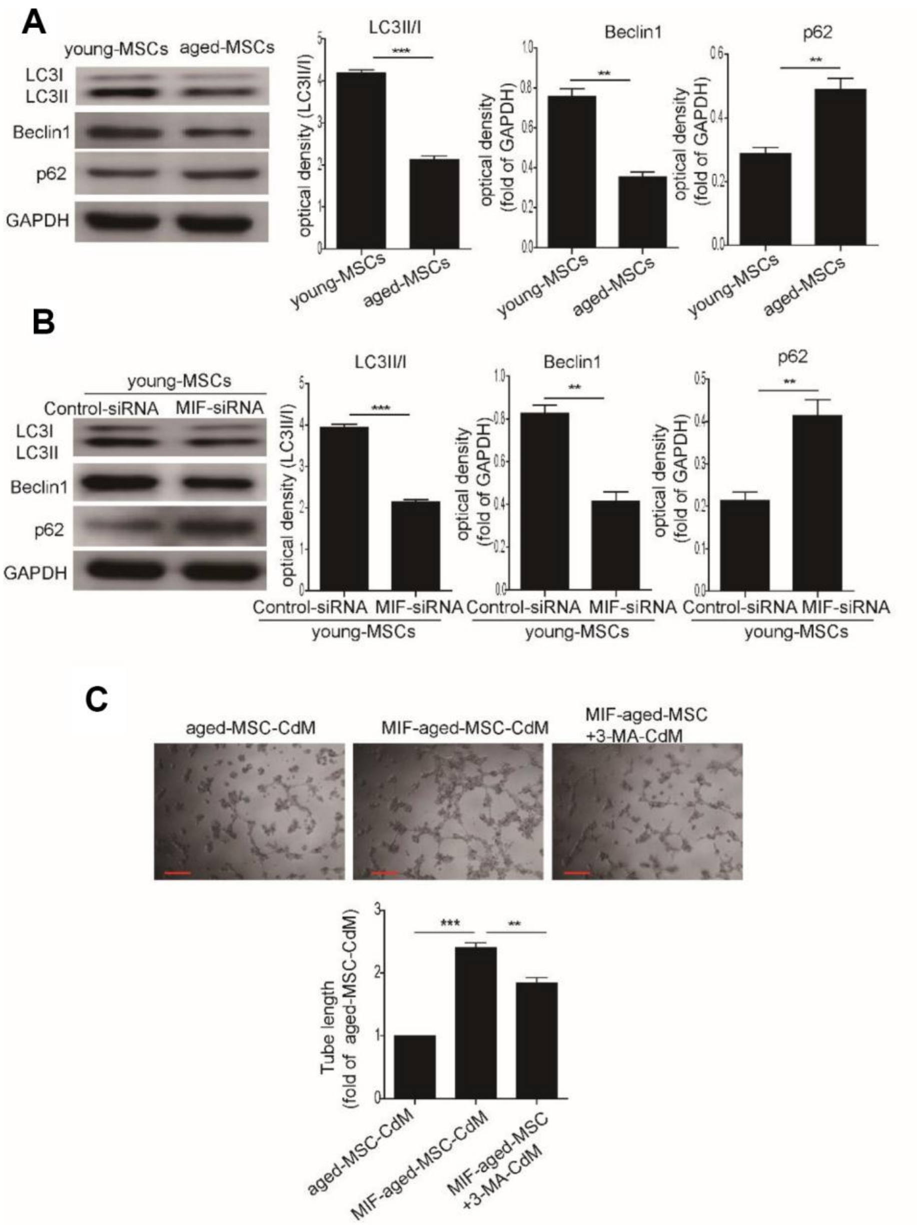


**Supplementary Figure 5. MIF mediated MSC senescence and angiogenic activity by regulating autophagy**. (**A**) Western blotting and quantitative analysis of the expression of LC3II/I, Beclin and p62 in aged-MSCs and young-MSCs. (**B**) Western blotting and quantitative analysis of the expression of LC3II/I, Beclin and p62 in young-MSCs transfected with control-siRNA or MIF-siRNA. (C) Representative images of tube formation and analysis of tube length in HUVECs treated with aged-MSC-CdM, MIF-aged-MSC-CdM or MIF-aged-MSC+3-MA-CdM. Scale bar=200µm. Data are expressed as the mean±SEM. n=3. ***p<0.01; ***p<0.001.*

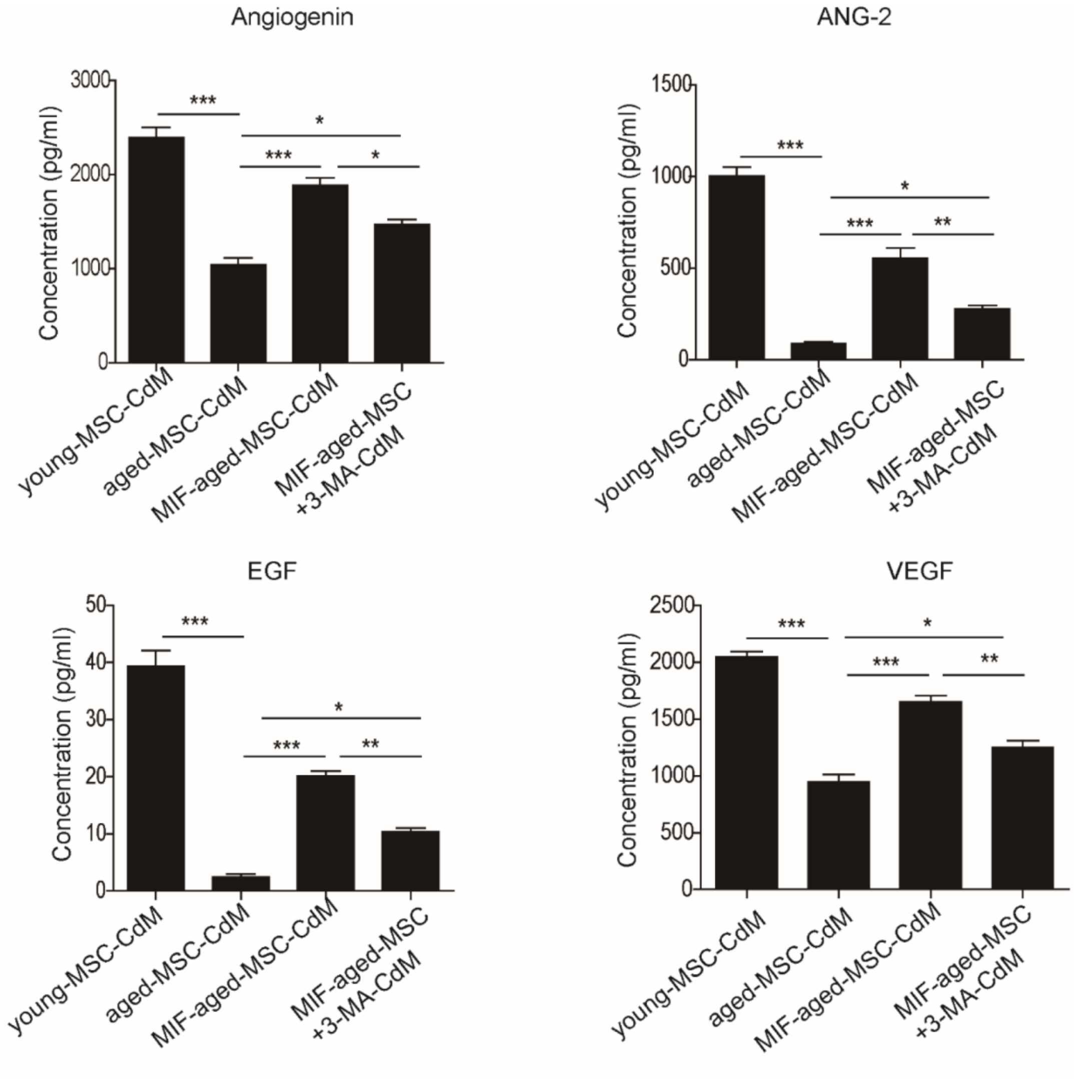


**Supplementary Figure 6. Antibody array analysis demonstrated alteration in the secretion of proangiogenic cytokines including Angiogenin, ANG-2, EGF and VEGF in young-MSC-CdM, aged-MSC-CdM, MIF-aged-MSC-CdM and MIF-aged-MSC+3-MA-CdM. Data are expressed as the mean±SEM.** n=3. *$p<0.05$; $^{**}p<0.01$; $^{***}p<0.001$.*
